# Honor to Whom Honor Is Due

**Published:** 1873-10

**Authors:** W. F. Fundenberg

**Affiliations:** Pittsburg, Pa.


### ARTICLE III.

### *Honor to Whom Honor is Due.*

BY W. F. FUNDENBERG, M. D., PITTSBURG, PA.

In the last edition of Dr. Garretson's "*System of Oral Surgery*," I was surprised to read a full and extended notice (occupying four pages) of Dr. S. P. Hullihen's great plastic operation performed upon Miss S——, of Newark, Ohio, without giving any credit whatever to the operator.

Surely if the operation was worthy of being qtuoed as an illustration of what plastic surgery is capable of doing in apparently hopeless cases,—the common courtesy due from one gentlemen to another required that the author's name should at least be given.

That Dr. Hullihen has been called from his labors by the stern messenger who will soon call all of us to our account, and that he is therefore unable to protect his reputation, does not make the matter any better. And while upon this subject, permit me to refer to another matter in justice to Dr. Hullihen. Dr. Garretson, in speaking of pressure applied to bring the jaw together in fissure of the hard palate, remarks that he had supposed that he (Dr. G.) was the originator of the idea himself, but states that he found in the "*Cosmos*," an extract taken from the "*Australian Medical Record*," in which the writer says that the plan was first presented to his mind in 1851.

Now it can easily be proven that Dr. Hullihen in 1841 suggested the idea of bringing the jaw together by pressure, (his method having been by adhesive straps,) and published his ideas in pamphlet form and in the journals. And although Dr. Hullihen called attention to the necessity of applying pressure during infancy, (the earlier the better in all cases of hare-lip with fissure of the jaw,) not only to prevent deformity, but also to simplify the operation upon the lip. I am surprised to see that not one surgeon in a hundred ever thinks of this method, so easily applied, and so excellent in its results, but suffers the patient to grow to adult years, and then attempt to remedy the defect with an obturator.

